# Ultrastructural Insight into Rift Valley Fever Virus Pathogenesis in Different Human Cell Types

**DOI:** 10.3390/ijms26178183

**Published:** 2025-08-23

**Authors:** Daniele Lapa, Maria Anele Romeo, Leonardo Duca, Carlotta Castelli, Eliana Specchiarello, Fabrizio Maggi, Laura Falasca

**Affiliations:** 1 Laboratory of Virology and Biosafety Laboratories, National Institute for Infectious Diseases Lazzaro Spallanzani—IRCCS, 00149 Rome, Italy; daniele.lapa@inmi.it (D.L.); mariaanele.romeo@inmi.it (M.A.R.); eliana.specchiarello@inmi.it (E.S.); fabrizio.maggi@inmi.it (F.M.); 2 Laboratory of Electron Microscopy, National Institute for Infectious Diseases Lazzaro Spallanzani—IRCCS, 00149 Rome, Italy; leonardo.duca@inmi.it (L.D.); carlotta.castelli@inmi.it (C.C.)

**Keywords:** RVFV, arbovirus, electron microscopy, pathogenesis, virus assembly and release, virus–host interaction

## Abstract

Rift Valley Fever Virus (RVFV) is an arbovirus that predominantly affects sheep, goats, and cattle, causing epizootics in livestock and epidemics in humans. Infection in pregnant livestock leads to high abortion rates and neonatal mortality. In humans, RVFV usually causes a self-limiting febrile illness, but severe forms can develop, such as hepatitis, hemorrhage, encephalitis, and death. In addition, the association between RVFV infection during pregnancy and miscarriages or stillbirths has been documented. RVFV is transmitted by a range of mosquito species, and, due to the diffusion of these insects, the virus has spread in several world regions, making possible the risk of a public health emergency. Nevertheless, research remains limited and cellular pathology is still poorly characterized. This work aimed to fill some knowledge gaps on the comprehension of RVFV pathogenesis. For this purpose, transmission electron microscopy (TEM) was used to analyze cellular modifications associated with RVFV morphogenesis in four human cell lines (HuH-7, LAN-5, A549, and HTR-8/SVneo) derived from liver, brain, lung, and placenta. Our results showed that all four cell lines are permissive to RVFV infection and highlighted differences in the cytopathogenesis associated with the cell type. These findings could have important implications in understanding disease mechanisms and developing antiviral strategies.

## 1. Introduction

RVFV is an arbovirus member of the Phlebovirus genus, within the Phenuiviridae family and Bunyavirales order, with high pathogenicity in ruminants and humans, which is emerging worldwide [1,2]. RVFV is transmitted by Aedes and Culex mosquito bites, and, because of the diffusion of these insects in large geographic areas, the virus has spread in several world regions, making possible the risk of health hazard in non-epidemic countries [3]. However, most human cases contract RVFV by coming into contact with infected animal fluids or tissues [4,5]. RVFV primarily infects domestic sheep, goats, and cattle, causing high mortality rates among young animals and “abortion storms” in pregnant domestic ruminants and camels. Studies have demonstrated that placental necrosis is responsible for fetal mortality in about 20% of ovine [6].

In humans, RVF is usually a self-limiting febrile illness, but some cases develop more severe manifestations such as liver damage, encephalitis and neurological disorders, hemorrhagic disease, and death [7]. In addition, as in livestock, the association between RVFV infection in women during pregnancy and an increased risk of spontaneous abortion has been established [8,9].

RVFV is an enveloped, negative-sense single-stranded RNA virus that has a tri-segmented RNA genome. The viral particles are spherical with a mean diameter of 90–110 nm; the surface of the envelope bears glycoproteins forming surface subunits that are regularly arranged [10]. RVFV replication occurs in the cytoplasm, and viral proteins are translated on free ribosomes or in the endoplasmic reticulum; the Golgi compartment appears the site for assembly of new viral particles. Nevertheless, the mechanisms of RVFV production are complex and still poorly characterized [11,12].

Despite having hepatocytes as primary targets, causing severe liver damage in both animals and humans [13], RVFV cellular tropism is very broad. Both in natural and experimental models, RVFV has demonstrated that, besides liver, it can infect many organs and cell types, including kidney, spleen, and lungs, as well as neurons and mononuclear phagocytic cells, giving a high variability of symptoms after infection [14,15]. The lungs are not a typical target for RVFV, but the virus can also be transmitted by aerosol exposure, and, after inhalation, lung tissue infection and neuropathology have been reported [16,17]. Studies also showed the ability of this virus to infect trophoblast cell lines [18,19], suggesting a possible route of transmission from the maternal blood to the fetal circulation.

Concerning organ damage, different mechanisms are thought to contribute to the pathogenesis caused by RVFV; thus, lesions in the liver appear caused by degeneration and massive necrosis of hepatocytes, due to the direct effect of the virus, while encephalitis (that occurs at later time) seems mediated mainly by immunopathological reaction [20]. Nevertheless, there are still knowledge gaps in the understanding of the RVFV life cycle and pathogenesis. Ultrastructural descriptions of RVFV replication sites and mechanisms of damage in different human tissues are scant; in particular, the pathogenesis of RVFV in human placental cells is still not clarified. The detailed characterization of viral particle production, propagation, and cell compartment remodeling may provide key elements to counteract the disease. To this aim, in this study, TEM was used to investigate, RVFV morphogenesis and associated host cell modifications in different human cell types.

## 2. Results

### 2.1. RVFV Is Able to Infect Human HuH-7, LAN5, A549, and HTR-8/SVneo Cell Lines and to Establish a Productive Infection

**Figure 1:**
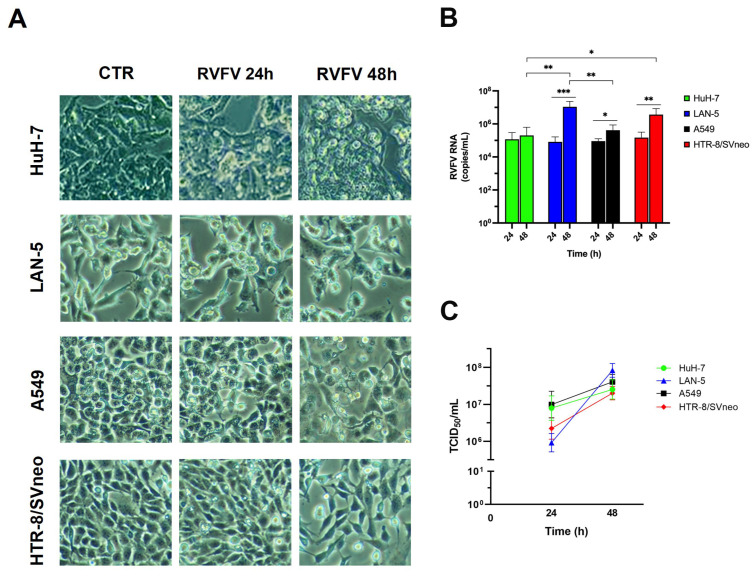
Susceptibility of human cell lines originating from different tissues to RVFV infection. (**A**) Four different cell lines (HuH-7, LAN-5, A549 and HTR-8/SVneo) were infected with RVFV at a MOI of 0.1; representative images of uninfected (CTR) and RVFV infected cells at 24 h and 48 h are shown. Original microscope’s magnifications: 20× (**B**) Quantitative RT-PCR evaluated in the different cell lines at 24 and 48 h p.i. (**C**) Replication kinetics calculated in the different cell lines at 24 and 48 h p.i. by TCID_50_ assay. Results are expressed as mean ± S.D of at least two independent experiments, with three biological replicates represented with 95% confidence intervals. Statistical analysis was performed using *t*-test and one-way ANOVA test; statistically significant difference is shown (* *p*-value < 0.05, ** *p*-value < 0.01, *** *p*-value < 0.001).

To evaluate the permissiveness of different human cell types to RVFV infection, we infected four cell lines (HuH-7, A549, LAN-5, and HTR-8/SVneo) with RVFV at a multiplicity of infection (MOI) of 0.1. All four cell lines were susceptible to RVFV infection. We analyzed the cytopathic effects (CPE) appearance and frequency by observing the proportion of infected cells exhibiting morphological changes. Visible changes in our system mainly consisted of loss of adhesion and cell rounding. Differences between the cell types consisted in the extent of changes and specific cellular alterations. At 24 h post infection (p.i.) only HuH-7 displayed a small proportion of cell rounding, while after 48 h about 50% of the cells appear round (Figure 1A) and with vacuoles inside (Supplementary Figure S1A).

Rounding of cells and vacuolization was also observed at 48 h p.i. in A549 cells, in about 10% of the cells with a focal distribution (Figure 1A, Supplementary Figure S1C). A total of 3–5% of HTR-8 cells at 48 h p.i. showed membrane blebs (Supplementary Figure S1D) while LAN-5 cells displayed less visible signs of cytopathic effects (Figure 1A, Supplementary Figure S1B).

The quantification of viral RNA produced by each cell type, using Real Time Reverse Transcription Polymerase Chain Reaction (Real Time RT-PCR) measurements, showed significantly higher levels at 48 h p.i. compared to 24 h p.i., except for the HuH-7 cells. (Figure 1B). At 48 h p.i., statistically significant lower RNA levels were observed in HuH-7 cells compared to LAN-5 and HTR-8/SVneo cells, and in A549 cells compared to LAN-5 (Figure 1B). RVFV titration, measured by the Tissue Culture Infectious Dose 50 (TCID_50_) assay, demonstrated comparable levels (<0.5 Log of difference) in all cell lines at 48 h p.i. (Figure 1C). This could be due to differences in packaging efficiency of infectious virus particles despite efficient expression.

### 2.2. Ultrastructural Analysis of RVFV Infection

To determine the pathology of RVFV infection and compare the intracellular modifications associated with viral morphogenesis, HuH-7, A549, LAN-5, and HTR-8/SVneo cells were infected for 48 h and analyzed by TEM. To evaluate infection-induced changes, the uninfected control cells in each cell line (Supplementary Figure S2) were examined in parallel.

**Figure 2:**
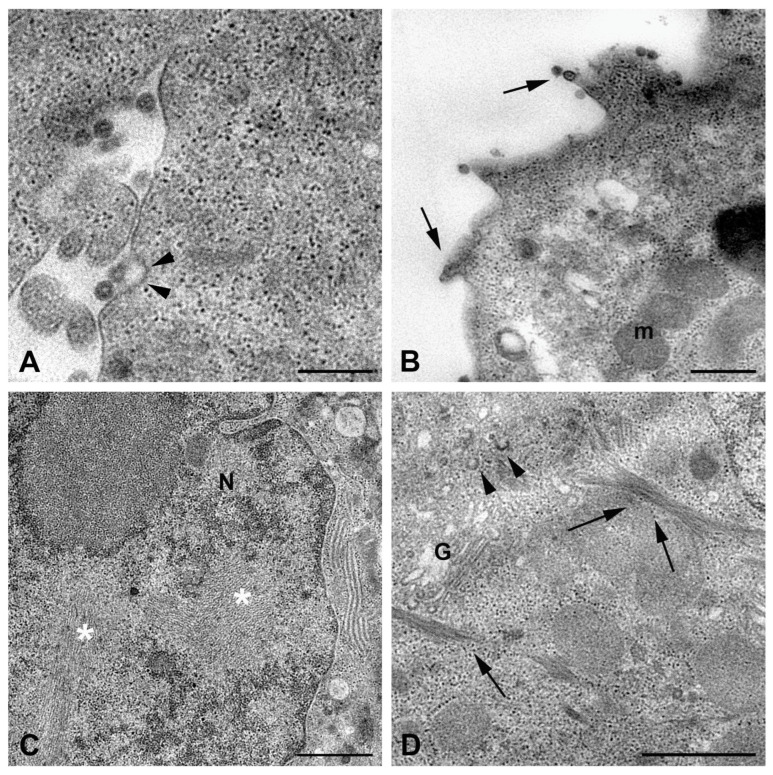
Transmission electron micrographs of RVFV-infected HuH-7 cells. Representative images of cells after 48 h of infection. (**A**) High magnification of HuH-7 cell surface shows entry of RVFV through invagination of thickened region of the plasma membrane (arrowheads). (**B**) Several viral particles are visible budding at the cell plasma membrane (arrows). (**C**) Large fibrillar aggregates (asterisks) formed in the nucleus (N) by filaments of RVFV NSs protein. (**D**) The image shows the presence of bundles of cytoskeletal filaments (arrows) in the cytoplasm of an infected cell; dilated sacks of Golgi complex (G), viral replication structures, and the maturation arcs (arrowheads) are visible. N, nucleus; m, mitochondria; G, Golgi complex. Scale bars: in (**A**,**B**,**D**) = 500 nm; in (**C**) = 1 µm.

**Figure 3:**
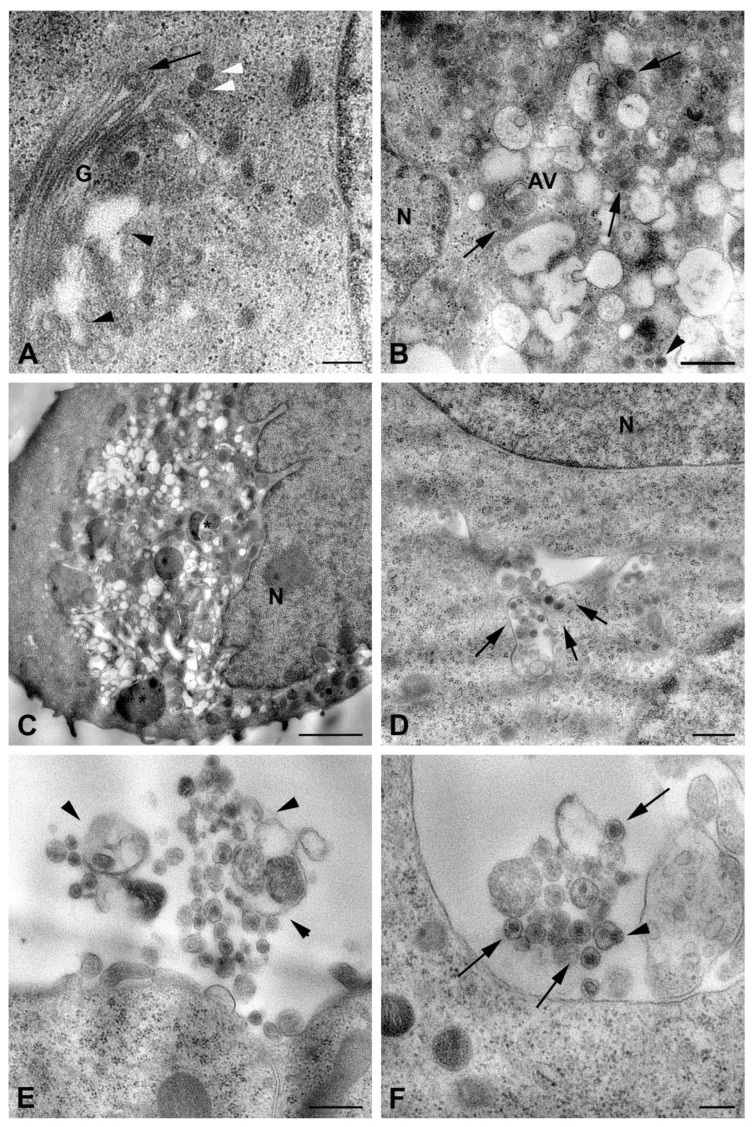
Ultrastructural features of RVFV replication in HuH-7 and LAN-5 cell lines. (**A**–**C**) Representative images of HuH-7 cells infected for 48 h with RVFV: (**A**) high magnification of Golgi complex (G) shows dilated cisternae (black arrowheads) in close proximity to viral particles with an annular-like morphology (arrow) and dense spherical particles (white arrowheads). (**B**) Viral factory in the cell cytoplasm near the nucleus (N). Many assembled viral particles are associated with small membrane compartments (arrows); a virion budding from one of the numerous vesicles present in this area is shown (arrowhead). (**C**) Vacuolization and cell rounding caused by RVFV in HuH-7 cells; phagolysosomes are visible (asterisks). (**D**–**F**) Representative images of LAN-5 cells infected for 48 h with RVFV: (**D**) viral particles enclosed in intracellular vesicles close to the plasma membrane (arrows). (**E**) Cluster of released viral particles associated with membrane vesicles (arrowheads). (**F**) Most extracellular viral particles are enclosed in small vesicles each containing only one particle (arrows), and image suggestive of virus budding from inside a vesicle is shown (arrowhead). G, Golgi complex; N, nucleus; AV, autophagic vacuole. Scale bars: in (**A**,**F**) = 200 nm; in (**B**,**E**) = 400 nm; in (**C**) = 1 µm; in (**D**) = 500 nm.

The liver represents one of the major targets of RVFV; thus, being the hepatocyte tropism well-recognized, we first examined ultrastructural features of RVFV infection in the HuH-7 hepatoma cell line (Figure 2 and Figure 3). Entry of the virus into cells likely appeared mediated by endocytic mechanism, as suggested by the detection of viral particles in invaginating thickened regions of the cell surface (Figure 2A).

Along the plasma membrane, many viral particles were observed budding from the surface (Figure 2B). Two kinds of modifications related to the infection were recognized: the presence of aggregates of filamentous structures in the nucleus (Figure 2C), as previously described in RVFV-infected mice [21], and the occurrence of bundles of cytoskeletal filaments in the cytoplasm (Figure 2D) [22]. In cells in which viral replication was less abundant, the assembly of virions was visible in association with the Golgi compartment, similarly to what was described for Bunyamwera virus (BUNV) [23]. The newly formed particles display different morphologies, appearing as dense viral particles with a more homogeneous appearance and annular-like particles, with a dark periphery and less dense center (Figure 3A).

Other structures intermediate of the viral maturation process, such as the maturation arcs, were also detected in close proximity of the Golgi complex (Figure 2D). However, in most of the cells, viral factories appeared as cytoplasmic areas filled with potential virus particles at different stages of maturation (with variability in the electron density) and associated with vesicular compartments, of which it was not possible to determine whether they originated from the Golgi apparatus (Figure 3B). A great number of membrane vesicles, which could represent dilatation of the endoplasmic reticulum cisternae, were present in the same area (Figure 3B,C), and viral particles were also found in association with them (Figure 3B). This massive viral replication was accompanied by severe cytopathic effects, consisting of the rounding of cells and extensive vacuolization of the cytoplasm (Figure 3C). The presence of autophagic vacuoles (Figure 3B) and phagolysosomes was also observed (Figure 3C).

The described cytopathic modifications occurring in infected HuH-7 cells were not found in infected LAN-5 neuroblastoma cells. The cytoplasm of LAN-5 cells at 48 h p.i. did not show vacuolization or other drastic morphological changes (Figure 3D). Rarely budding virions from the LAN-5 cells surface were observed; in contrast, most viral particles in the extracellular environment were associated with membrane vesicles (Figure 3E,F). Indeed, large vesicles containing viral particles were found in the cytoplasm close to the plasma membrane (Figure 3D), suggesting an exocytosis mechanism for virus release. However, most of the extracellular viral particles were observed as single particles enclosed within small vesicles, likely exosomes (Figure 3E,F). Particles inside these vesicles showed heterogeneous features, some of them being more electron dense, while others were suggestive of virions’ packaging taking place directly inside the vesicles (Figure 3F).

**Figure 4:**
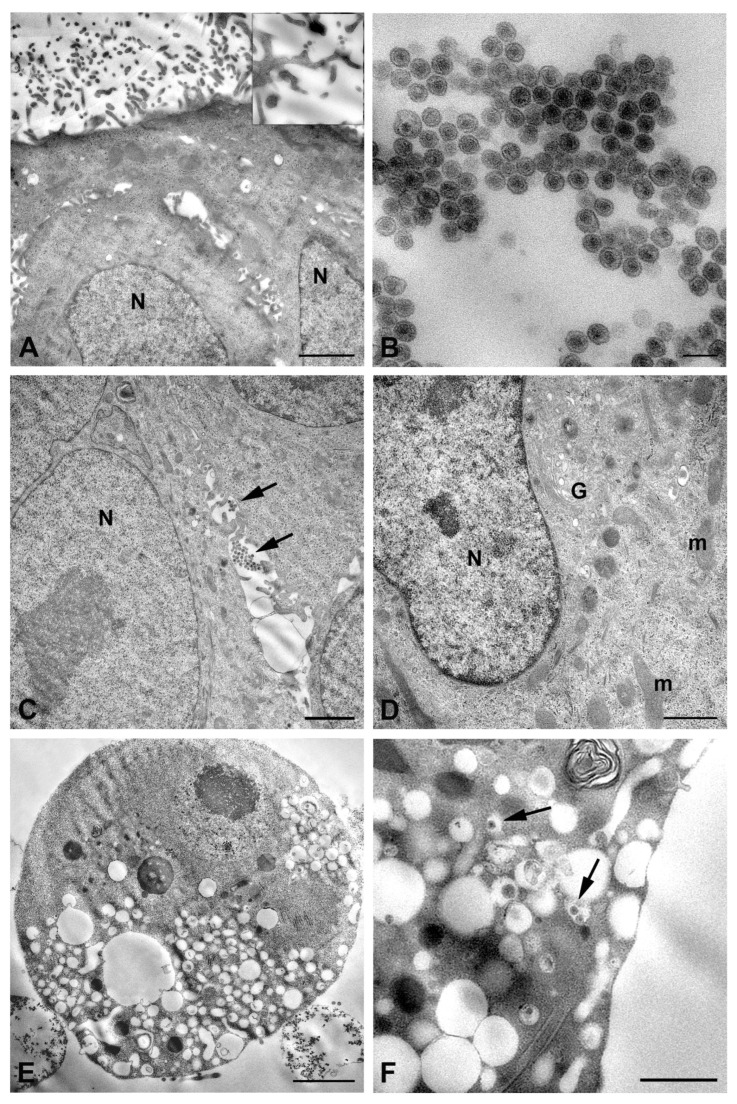
Transmission electron micrographs of RVFV-infected A549 cells. Representative images of cells after 48 h of infection. (**A**) Numerous viral particles are visible along plasma membrane extroflessions. The insert shows virus budding from the surface of microvillous projections; (**B**) high magnification of one of the virus clusters detected outside the cells; (**C**,**D**) infected cells without evident nuclear and cytoplasmic organelle modifications are shown. Arrows in (**C**) point to viruses close to the cells surface; (**E**) cytopathic effect of viral infection causes rounding and vacuolization of the cell; (**F**) RVFV particles (arrows) are present in the lumen of cytoplasmic vacuoles. N, nucleus; m, mitochondria; G, Golgi complex. Scale bars: in (**A**,**C**,**E**) = 2 µm; in (**B**) = 200 nm; in (**D**,**F**) = 1 µm.

In RVFV-infected A549 cells, which were chosen as a human alveolar epithelial cells model, electron microscopy observation showed that the release of viral particles occurred by budding at the cell surface (Figure 4A), particularly along the cytoplasmic extroflections (Figure 4A, insert).

Extracellular virions displayed homogeneous morphology and were usually found in clusters (Figure 4B). Despite the release of numerous virions, the majority of A549 cells at 48 h p.i. did not display evident signs of cytopathogenesis as concern both nucleus and cytoplasmic organelles (Figure 4C,D). On the contrary, other cells showed strong cytopathic effects, resulting in cell rounding and vacuolization of the cytoplasm (Figure 4E). In these cells, viral particles were observed within the enlarged vacuoles (Figure 4F).

**Figure 5:**
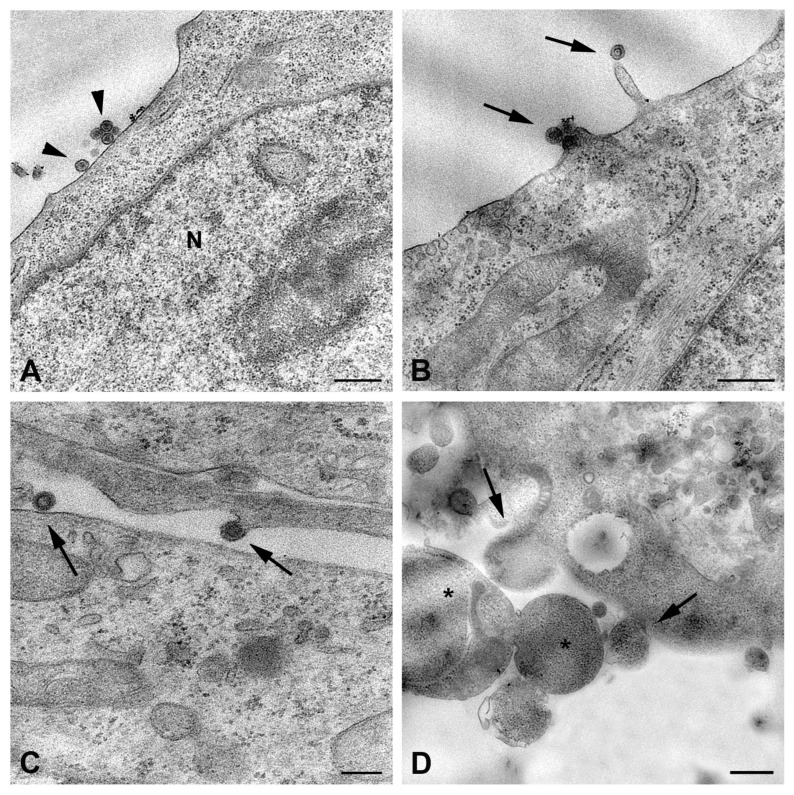
Transmission electron micrographs of RVFV infection in HTR8/SVneo cells. Representative images of cells after 48 h of infection. (**A**) A small group of viruses is visible at the cell surface (arrowheads); (**B**) viral particles are released by budding from the plasma membrane (arrows); (**C**) newly released particles display homogeneous morphology and clear evident layer of spikes (arrows); (**D**) cytopathic effect of viral infection: blebbing of the cell membrane (arrows) and release of apoptotic bodies (asterisks) are shown. N, nucleus. Scale bars: in (**A**,**B**,**D**) = 500 nm; in (**C**) = 200 nm.

To explore the susceptibility of the placental cells to RVFV infection, HTR8/SVneo cells were used as a closer model of trophoblast cells. Electron microscopy analysis confirmed that these cells were permissive to the infection and sustained the replication, and they revealed some differences compared to RVFV infection in the other cell types examined. Egress of virions was found singly or in small groups (Figure 5A,B).

**Figure 6:**
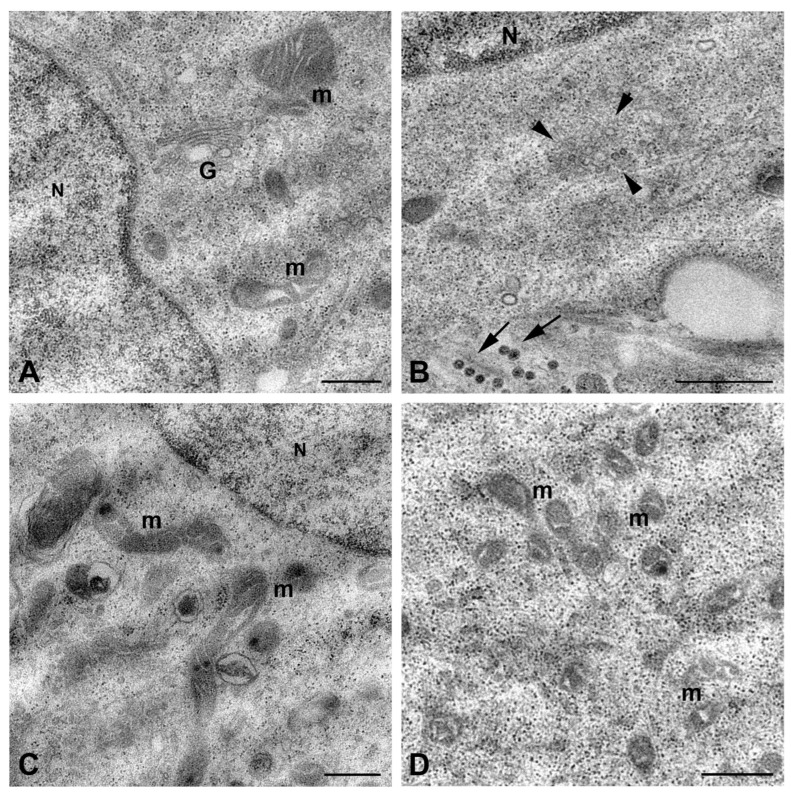
Ultrastructural features of HTR8/SVneo cells after infection with RVFV. Representative images of cells after 48 h of infection. (**A**) Golgi cisternae are visible near the nucleus; (**B**) vesicular structures (arrowheads), likely representing viral replication sites, are present in the perinuclear area. Arrows point to mature viral particles; (**C**,**D**) images show altered mitochondrial architecture, with inner membrane distortion and bulging or swirling cristae. N, nucleus; G, Golgi complex; m, mitochondria. Scale bars: in (**A**,**C**,**D**) = 500 nm; in (**B**) = 1 µm.

Viral particles were released by budding at the cell plasma membrane; they appeared homogeneous in shape, displaying an internal dense core surrounded by an envelope in which the layer of spikes is clearly recognizable (Figure 5B,C). Some cells displayed cell blebbing suggestive of apoptotic cell death (Figure 5D); however, the majority of HTR8/SVneo cells at 48 h p.i. did not show RVFV-induced cytopathic effect. No clear evidence of Golgi apparatus involvement in viral morphogenesis had been observed (Figure 6A), although perinuclear cytoplasmic areas rich in small vesicular structures were observed, suggesting they could represent sites of viral replication.

On the contrary, mitochondria appeared strongly affected, exhibiting striking modifications of their structure, which consisted of fragmentation and inner membrane distortion with bulging or swirling cristae (Figure 6C,D).

## 3. Discussion

RVFV is a major emerging arbovirus of significant medical and veterinary importance. Due to the lack of a licensed vaccine for human use, the World Health Organization (WHO) has prioritized RVFV among high-risk pathogens requiring urgent research to address critical gaps in understanding its mechanisms of infection [24].

The comprehension of the pathogenesis of a viral infection is strictly linked to unraveling virus tropism and host–cell interaction. This study aimed to investigate the ultrastructural features of RVFV morphogenesis in human cell lines derived from various tissues. The results revealed cell type-dependent differences in viral replication, morphogenesis, and virus–host interactions.

Previous studies, including post-mortem analyses from the 1977 Egypt outbreak and experimental animal models, have shown that RVFV can disseminate across multiple tissues [25,26]. However, discordance between immunohistochemical detection of viral components and electron microscopy findings suggest the possibility of abortive infections and indirect tissue damage mediated by the host’s pro-inflammatory cytokine response, rather than direct cytopathic effects [21]. Despite the great number of species belonging to the Bunyavirales order, their virus life cycles share similarities [27] and, as far as we know, the assembly of new viruses occurs at the Golgi complex by budding of viral particles into the lumen of the Golgi vesicles [23,28]. In our experiments, this process was clearly observed only in HuH-7 hepatic cells. Additionally, in these cells, virions were also seen budding directly at the plasma membrane. RVFV particles were also found at the cell surface in the A549 (lung epithelial) and HTR-8/SVneo (placental trophoblast) cell lines. Moreover, under conditions of extensive cytoplasmic vesiculation, budding virions were detected within the lumen of cytoplasmic vesicles in both HuH-7 and A549 cells. These findings suggest that RVFV has the ability to take advantage of multiple cellular membrane systems, which can vary based on the cell type or on the state/condition of the host cell.

A particularly noteworthy observation was made in LAN-5 neuroblastoma cells, where RVFV particles were detected within extracellular vesicles (EVs). While this mode of viral dissemination has been described for other enveloped and non-enveloped viruses [29], it has not been previously reported for RVFV. Inclusion within EVs may facilitate immune evasion [30] and enhance infectivity through local viral concentration, thereby elevating the multiplicity of infection [31]. This mechanism assumes relevance for the infection of less permissive cells. For example, Zika Virus (ZIKV) utilizes EV release from astrocytes to spread into the nervous system and infect neurons which were less susceptible to ZIKV infection [32,33]. Given that RVFV-induced central nervous system pathology typically arises in advanced stages of infection, our observation that RVFV packaging occurs within EVs in LAN-5 cells supports a potential mechanism for neuroinvasion. Furthermore, in our experimental conditions, RVFV was not only transported within EVs but also appeared to undergo assembly inside these vesicles, suggesting a potential role for EVs as carriers of viral RNA and proteins.

A peculiar feature of RVFV infection is the amyloid structures in the nucleus made by filaments of the NSs protein of RVFV [34]. These fibrillar structures are critical for antagonizing the host’s innate immune responses [35]. The presence of nuclear NSs fibrils has been reported by other authors in various mammalian cell lines at early times p.i. (4–16 h); however, the level of NSs in the nucleus appeared dependent on the MOI of infection [35,36]. In our study, these amyloid aggregates were exclusively observed in the nuclei of HuH-7 cells. This observation may be explained by the lower MOI used (0.1) compared to the higher MOIs (>0.5) applied in previous studies. Our analysis was conducted only at 48 h p.i., raising questions about the persistence of these aggregates over time. Interestingly, disappearance of nuclear NSs fibrils has been observed in mosquito cells at later stages of infection [37].

Our study also provides, to our knowledge, the first evidence that RVFV infection induces ultrastructural mitochondrial alterations in HTR-8/SVneo placental trophoblast cells. Mitochondria are dynamic organelles responsible for cell energy production but are also involved in many pivotal cell pathways, such as cell death, calcium homeostasis, and generation of reactive oxygen species (ROS). In addition, mitochondria play an important function in immunity, producing mitochondrial damage-associated molecular patterns (DAMPs) and mitochondrial antiviral signaling protein (MAVS) [38]. Mitochondrial architecture is dynamically regulated through fusion and fission processes, which are tightly linked to functional status [39]. Morphological changes are indicative of altered mitochondrial function; fragmented mitochondrial morphology is a well-established indicator of oxidative stress and mitochondrial dysfunction [40]. In RVFV-infected HTR-8/SVneo cells, mitochondria displayed swollen cristae and reduced size, consistent with increased mitochondrial fission and possible ROS overproduction. A number of recent studies focused attention on a possible association between mitochondrial dysfunction and placental disorders and pregnancy loss. Excessive ROS production and oxidative stress-related damage have been observed in most studies concerning preeclampsia (PE) and fetal growth restriction (FGR) [41]. On this basis, our findings lead us to speculate on a possible mechanism to be investigated in order to explain the increased risk for miscarriage in RVFV-infected pregnant women [8].

Our data expand current knowledge regarding the pathogenic effects of RVFV replication. However, this study still has some limitations: the lack of functional assays, as concern viral EV-mediated release and mitochondrial damage, does not allow us to give definitive conclusions apart from this preliminary descriptive report of morphological features.

In conclusion, this work provides novel ultrastructural insight into cell modifications induced by RVFV in different human cell lines, and the results obtained highlighted differences associated with the host cell. These findings could have important implications for understanding RVFV pathogenesis and for therapeutic development.

## 4. Materials and Methods

### 4.1. Cell Lines

HuH-7 (Hepatocellular carcinoma; ATCC, Manassas, VA, USA), A549 (Lung cancer; ICLC Cell Bank, Genova, Italy), LAN-5 (Neuroblastoma; ATCC) and VeroE6 (African green monkey kidney; ATCC) were grown in Modified Eagle Medium (MEM), 10% Fetal Bovine Serum (FBS), L-glutamine (2 mM), streptomycin (100 μg/mL), and penicillin (100 U/mL) in 5% CO_2_ at 37 °C. HTR-8/SVneo (Trophoblast; kindly provided by Prof. Luisa Campagnolo) was grown in RPMI 1640, 10% Fetal Bovine Serum (FBS), L-glutamine (2 mM), streptomycin (100 μg/mL), and penicillin (100 U/mL) in 5% CO_2_ at 37 °C.

### 4.2. Viral Production

RVFV was obtained from the National Collection of Pathogenic Viruses (NCPV) and propagated in VeroE6. Confluent VeroE6 cells were washed twice with PBS 1X and infected with RVFV at a MOI of 0.5 TCID_50_/cell. After 1 h of adsorption, the viral inoculum was removed, and cells were cultured for 48 h. At 48 h p.i., the viral suspension was collected and clarified by centrifugation at 2000 rpm for 10 min. After three freezing/thawing cycles, it was aliquoted and stored at −80°C. Virus titers were determined by limiting dilution assay and residual infectivity was expressed as TCID_50_/mL, calculated according to the Reed and Muench method. Viral production was performed in a Biosafety Level 3 (BSL-3) laboratory.

### 4.3. Viral Infection

RVFV produced in VeroE6 and titred by TCID_50_/mL assay was used for the following infections. HuH-7, A549, LAN-5, and HTR-8/SVneo cell lines were plated on 8-well chamber slides (Fisher Scientific, Segrate (MI), Italy) at a density of 8 × 10^4^ cell/wells. The day after, cells were infected with RVFV at a MOI of 0.1 TCID_50_/cell. After 1 h of adsorption, the viral inoculum was removed, and cells were cultured for 48 h. Uninfected cells were used as mock control. After 48 h of infection, the viral suspension was recovered to measure the viral titer. Viral infection was performed in BSL-3.

### 4.4. TCID_50_ Assay

To measure RVFV titer in the different cell lines at 48 h p.i., a TCID_50_ infectivity assay was performed. Briefly, VeroE6 cells were seeded in 96-well tissue culture plates at a density of 1 × 10^4^ cells/well in a growth medium containing 10% FBS and incubated at 37 °C in a humidified atmosphere with 5% CO_2_. The day after, semi-confluent cell monolayers were infected with serial dilutions of viral suspension obtained by the different cell lines, in a growth medium containing 2% FBS (four replicates were analyzed for each dilution) and incubated at 37 °C. The virus-induced CPE was evaluated at six days p.i.

### 4.5. Viral Quantification by Real-Time RT-PCR

Nucleic acids were extracted from the supernatant of the different cell lines at 48 h p.i. using QIAamp^®^ Viral RNA (QIAGEN, Hilden, Germany) according to manufacturing instructions (Qiagen RNeasy Mini Handbook online; https://www.qiagen.com/us/resources/resourcedetail?id=14e7cf6e-521a-4cf7-8cbc-bf9f6fa33e24&lang=en (accessed on 7 June 2023)). RVFV RNA was detected by the commercial assay RealStar^®^ Rift Valley Fever Virus RT-PCR Kit 1.0 (Altona Diagnostics, Segrate (MI). Italy). Assay conditions were as follows: reverse-transcription at 55 °C for 20 min, denaturation at 95 °C for 2 min, then 45 cycles of 95 °C for 15 s, 55 °C for 45 s, 72 °C for 15 s (Altona Diagnostics RealStar^®^ Rift Valley Fever Virus RT-PCR Kit 1.0. https://www.altona-diagnostics.com/en/products/reagents-140/reagents/realstar-real-time-pcr-reagents/realstar-rvfv-rt-pcr-kit-ce.html (accessed on 2 July 2023)). It has already been demonstrated that this RT-PCR method did not cross-react with Dengue Virus, Japanese Encephalitis Virus, St. Louis Encephalitis Virus, Usutu Virus, Marburg Virus, Ebola Virus, West Nile Virus, Yellow Fever Virus, or Zika Virus. The analytical assay sensitivity was 890 copies/mL. The precision data was determined by evaluating intra-assay, inter-assay, and inter-lot variability. The coefficient of variation of the test is 1.10% as indicated in the Altona brochure (Altona Diagnostics; https://altona-diagnostics.com/wp-content/uploads/2023/12/RealStar-RVFV-RT-PCR-Kit-1.0_WEB_RUO_EN-S02.pdf (accessed on 12 June 2023)).

The RVFV standard RNA, provided by Altona Diagnostics, was serially diluted to a theoretical range of 10^6^ to 10^−1^ copies to generate a standard curve. The interpolation of the data was performed using GraphPad Prism version 9 (GraphPad Software, La Jolla, CA, USA) using the Sigmoidal, 4PL model.

### 4.6. Transmission Electron Microscopy

HuH-7 cells, LAN-5 cells, A549 cells, and HTR-8/SVneo cells were infected with RVFV at a MOI of 0.1 and processed for TEM using standard procedures. Cells were seeded into 8-well chamber slides at a density of 8 × 10^4^ cells per well. After 48 h of infection, cells were washed with 0.1 M cacodylate buffer, fixed for 4 h with 2.5% glutaraldehyde in 0.1 M cacodylate buffer at 4 °C, and post-fixed with 1% OsO_4_ in the same buffer. Samples were then dehydrated in graded ethanol and embedded in Epon resin. Ultrathin sections were stained with 2% uranyl acetate and observed with a transmission electron microscope, JEOL JEM 2100 Plus (Japan Electron Optics Laboratory Co., Ltd., Tokyo, Japan). Images were digitally captured with a digital camera TVIPS (Tietz Video and Image Processing Systems GmbH, Gauting, Germany).

### 4.7. Statistical Analyses

Statistical analyses and graphical representations were performed using GraphPad Prism version 9 (GraphPad Software). Differences among the virus derived from the cell lines were assessed using one-way ANOVA, and Student’s *t*-test was used for specific pairwise comparisons. Differences were considered significant if the adjusted *p*-value was less than 0.05.

## Data Availability

The original contributions presented in the study are included in the article. Further inquiries can be directed to the corresponding author.

## Supplementary Materials

The following supporting information can be downloaded at: https://www.mdpi.com/article/10.3390/ijms26178183/s1.

## Abbreviations

The following abbreviations are used in this manuscript:

RVFV	Rift Valley Fever Virus
TEM	Transmission Electron Microscopy
NCPV	National Collection of Pathogenic Viruses
MOI	Multiplicity of Infection
TCID_50_	Tissue Culture Infectious Dose 50
p.i.	Post Infection
BSL-3	Biosafety Level 3
CPE	Cytopathic Effect
RT-PCR	Reverse Transcription Polymerase Chain Reaction
BUNV	Bunyamwera Virus
EVs	Extracellular Membrane Vesicles
ZIKV	Zika Virus
ROS	Reactive Oxygen Species
MAVS	Mitochondrial Antiviral Signaling Protein
DAMPs	Damage-Associated Molecular Patterns
PE	Preeclampsia
FGR	Fetal Growth Restriction
