# Supplementary material for: Ultrastructural Insight into Rift Valley Fever Virus Pathogenesis in Different Human Cell Types
Source: Int J Mol Sci. 2025 Aug 23;26(17):8183. doi: 10.3390/ijms26178183 (PMC12428719; doi:10.3390/ijms26178183)
Supplement: Supplementary file 1 [file ijms-26-08183-s001.zip › ijms-3723935-supplementary.pdf]

Supplementary Figure S1

"Ultrastructural insight into Rift Valley Fever Virus pathogenesis in different human cell types."

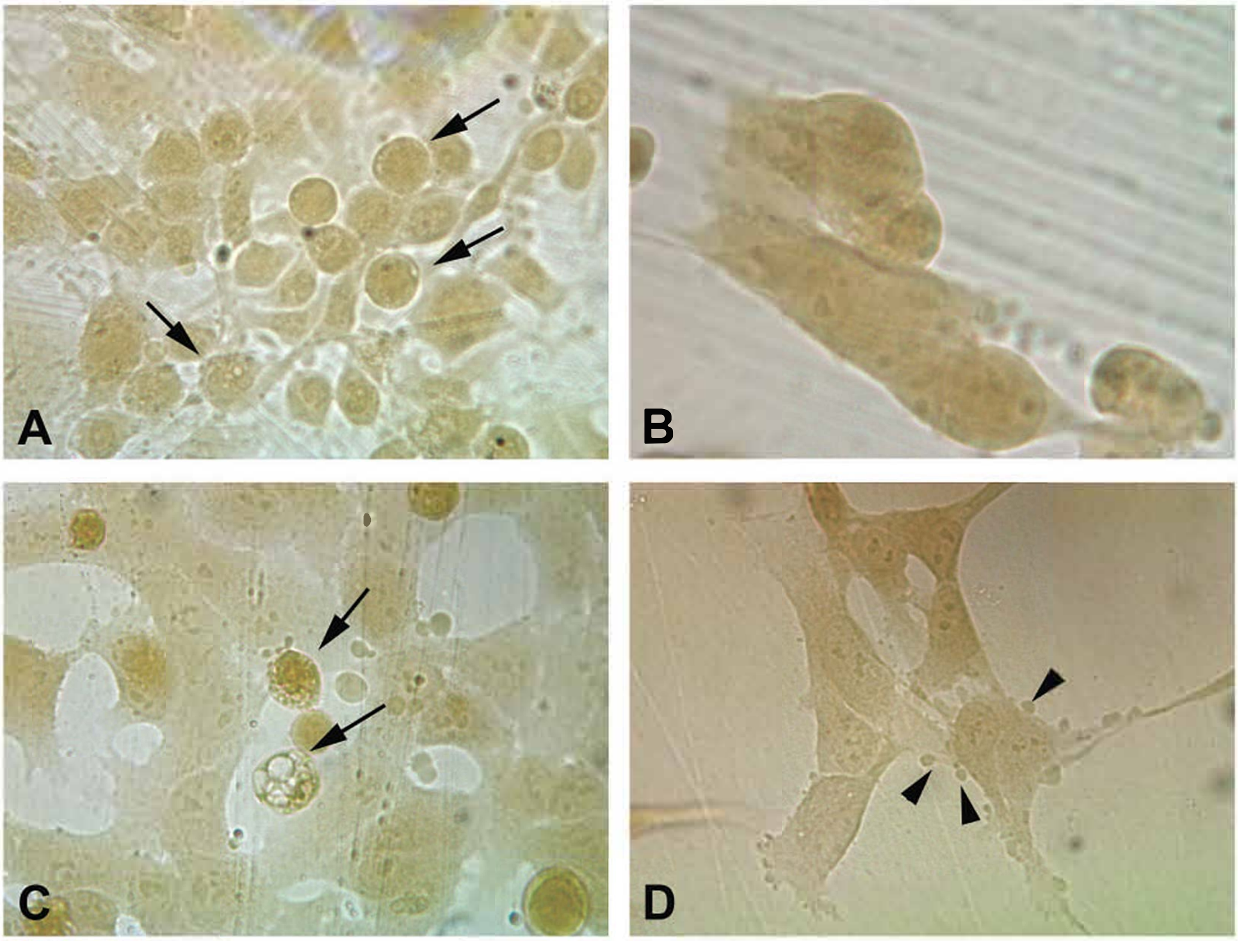

Representative photomicrographs of cell morphology in RVFV infectected HuH-7 (A), LAN-5 (B), A549 (C) and HTR-8/SVneo (D). at 48 h.p.i. Cells were fixed for electron microscopy preparation and observed using standard brightfield microscopy with the 40X objective.

Arrows point to round and vacuolized cells. Arrowheads indicate membrane blebs.

Supplementary Figure S2

"Ultrastructural insight into Rift Valley Fever Virus pathogenesis in different human cell types."

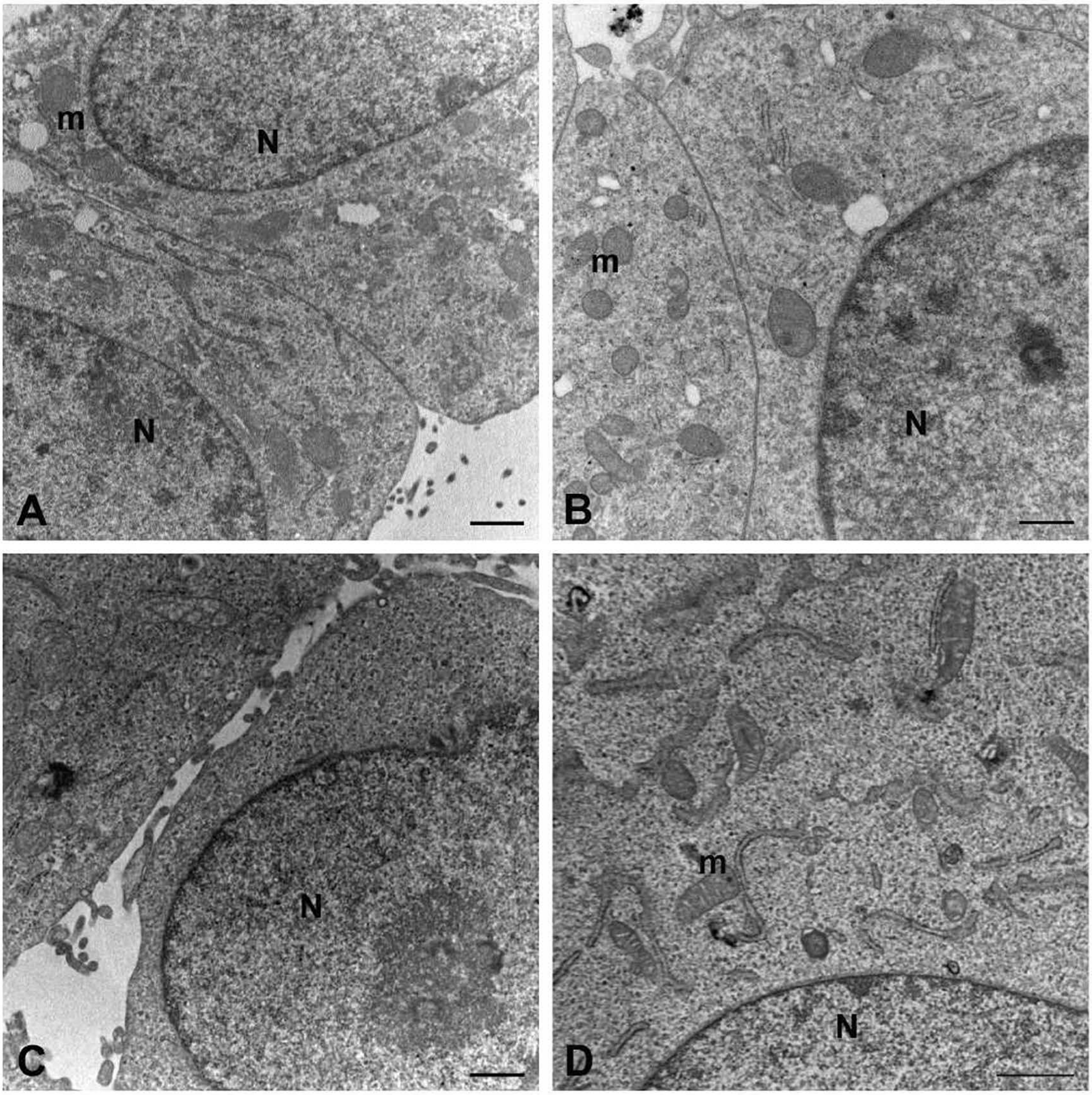

Representative transmission electron micrographs of uninfectected control HuH-7 (A), LAN-5 (B), A549 (C) and HTR-8/SVneo (D).

N, nucleus; m, mitochondria

Scale bars: A,B,C= 500nm; D =1μm
